# The Effect of Prehabilitation on Health Resource Use and 1‐Year Survival: An Observational Cohort Evaluation of the Active Together Service

**DOI:** 10.1111/jep.70425

**Published:** 2026-04-21

**Authors:** Kerry Rosenthal, Nik Kudiersky, Gabriella Frith, Gail Phillips, Carol Keen, Daniel Howdon, Shaun Barratt, Diana M. Greenfield, Gary H. Mills, Anna Myers, Liam Humphreys, Robert Copeland

**Affiliations:** ^1^ School of Sport and Physical Activity, Advanced Wellbeing Research Centre Sheffield Hallam University Sheffield UK; ^2^ Sheffield Teaching Hospitals NHS Foundation Trust Royal Hallamshire Hospital Sheffield UK; ^3^ Academic Unit of Health Economics University of Leeds Leeds UK

**Keywords:** cancer, dietetics, exercise, prehabilitation, psychology, surgical oncology

## Abstract

**Introduction:**

Research suggests that multi‐modal prehabilitation can improve quality of life and clinical outcomes. There is, however, limited evidence on the effect of prehabilitation on hospital resource use.

**Methods:**

This is a non‐randomised observational cohort evaluation. The intervention group were patients receiving multi‐modal prehabilitation (Active Together) before surgery for colorectal, lung or upper gastrointestinal cancer between January 2022 and March 2024. Patients who declined to participate in Active Together and historical patient data (2017–2021) were used as comparator groups. Outcome measures were length of hospital stay, length of critical care stay, total number of days spent in hospital as a readmission within 90 days following surgery, and one‐year survival rate.

**Results:**

Three hundred and five patients completed prehabilitation, 96 patients declined to join the service, and 869 patients were included in the historical dataset. Active Together colorectal patients spent less time in critical care than historical colorectal patients (0.9 vs 1.2 days, *p* = 0.011). Whereas Active Together lung patients spent longer in critical care than historical lung patients (2.5 vs 1.7 days, *p* < 0.001). One‐year survival rate was greater in Active Together patients compared to the declined group (95% vs 85%, *p* = 0.013) but did not differ significantly from the historical group (95% vs 92%, *p* = 0.140). The probability of prehabilitation being more cost‐effective than not receiving prehabilitation was 58%, 60%, and 59% for colorectal, lung and upper gastrointestinal patients, respectively.

**Conclusion:**

The impact of prehabilitation on healthcare resource use was mixed with promising evidence of a positive effect of prehabilitation and rehabilitation on overall survival. There were notable differences between tumour groups in these outcomes which warrants further investigation. Future research is needed to build on these findings by including a larger sample size, a wider range of tumour groups, and a longer follow up period.

## 1 Introduction

Surgeries for colorectal, lung or upper gastrointestinal (GI) cancers are invasive, and it can take patients many months, if at all, to return to their pre‐surgery functional ability [1]. There is also a high risk of surgery‐related complications, which can lead to increased mortality and morbidity [2, 3]. Prehabilitation aims to optimise patient resilience prior to surgery, improve postoperative outcomes, and facilitate recovery and increasingly involves a multi‐modal intervention combining physical activity, dietetic and psychological support [4, 5]. There is growing evidence that prehabilitation can improve clinical outcomes and quality of life [6, 7]. Additionally, prehabilitation may improve patient eligibility for surgery [8, 9, 10], allowing patients to access treatments otherwise unavailable to them. Despite evidenced benefits, prehabilitation is not standard care for cancer patients across the UK.

The length of stay post‐surgery is often used as an indicator of the patient's recovery and treatment costs. Reducing length of stay is part of the business‐case justification for investment in prehabilitation. However, it is a highly variable measure, as many factors can lead to a longer length of stay, such as comorbidities, clinical judgement, and day of admission [11]. A previous meta‐analysis showed that exercise interventions prior to surgery for lung cancer patients significantly reduced length of stay by an average of 4.2 days [12]. Similarly, a meta‐analysis found a 3.7‐day reduction in length of stay for abdominal cancer patients who received prehabilitation compared to controls [13]. The individual studies included in the meta‐analysis did not find significant differences in length of stay when analysed separately [14, 15, 16], indicating the need for large sample sizes to identify changes in this highly variable measure.

A recent study on prehabilitation prior to colorectal surgery showed an average reduction in hospital stay (0.91 days) and a cost saving of €140 per patient [17]. Two systematic reviews of studies examining prehabilitation cost show that generally, prehabilitation for surgery tends to be cost‐effective; however, they caution the risk of publication bias [18, 19].

Previous evidence suggests that prehabilitation improves disease‐free survival in colorectal cancer patients [20, 21]. However, the evidence in this area for other cancers remains limited [12, 22]. A three‐year rehabilitation programme for patients with colorectal cancer resulted in significantly improved eight‐year survival rates (90.3% survival compared to 83.2%), although limited differences were seen after one year.

Further insights into the effects of prehabilitation on length of stay, cost‐savings and survival are required to build on the limited evidence available. Additionally, prehabilitation in a real‐world setting, including the most complex patients, has not been fully explored.

Active Together is a prehabilitation and rehabilitation service in Sheffield, UK, that provides multimodal support for patients undergoing treatment for lung, colorectal or upper GI cancer [23, 24]. The service is integrated into the NHS cancer care pathway, and all eligible patients are referred to the service. This paper aims to estimate the effectiveness of the Active Together intervention compared to the no intervention comparators on one‐year survival and healthcare resource use. It is hypothesised that patients who received prehabilitation with Active Together will have a higher one‐year survival rate and lower healthcare resource use.

## 2 Methods

The protocol for this non‐randomised observational cohort evaluation has been previously reported [24]. A randomised‐control trial was not considered ethical, given the demonstrated clinical benefits of prehabilitation [6, 7]; therefore, this study aims to emulate a trial by a single arm observational study with appropriate comparator groups. This study has adopted a healthcare system perspective. This study has been reported in line with the Strengthening the Reporting of Observational Studies in Epidemiology (STROBE) checklist which is available in appendix 1.

### 2.1 Ethics Statement

The service evaluation was approved by the Clinical Effectiveness Unit at Sheffield Teaching Hospitals NHS Foundation Trust (Ref 11115 ‐ May 19, 2022). Evaluation data were pseudonymised prior to analysis to ensure patient confidentiality. Patients attending Active Together provided informed consent to share hospital data for the evaluation. Patients who did not attend Active Together were included if they had not opted out the NHS National Data Opt‐Out Service.

### 2.2 Data Sources

A list of Active Together participants and prehabilitation dates, and surgical data for all groups were provided by Sheffield Teaching Hospitals NHS Foundation Trust.

### 2.3 Participants

**Figure 1:**
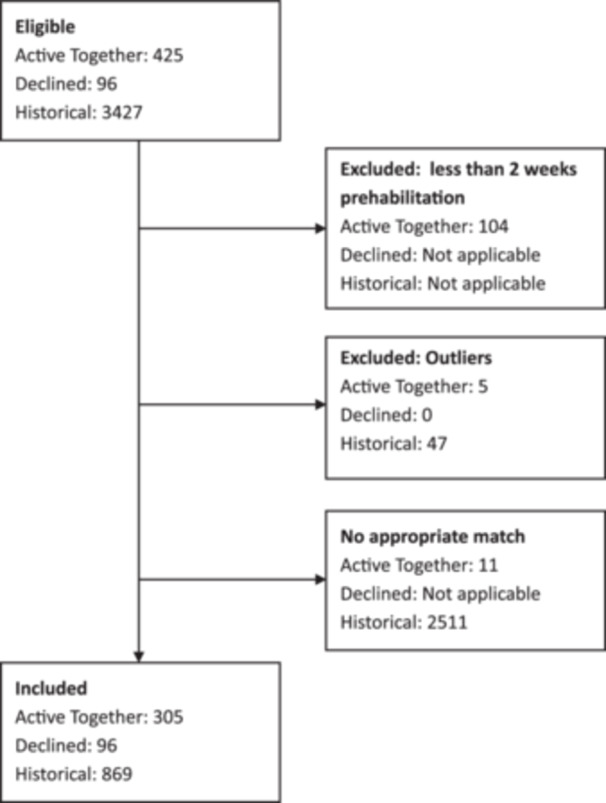
A flow diagram summarising patient numbers included at each stage.

All eligible patients within the evaluation time frame were included. Figure 1 shows a flow diagram summarising patient numbers included at each stage.

#### 2.3.1 Prehabilitation Group

Patients receiving curative treatment for colorectal, lung or upper GI cancer were referred to Active Together at the point of diagnosis. Active Together provided personalised physical activity, dietetic and psychological support, depending on patients' level of need, which was determined by a range of factors including existing comorbidities and baseline level of fitness. Patients with a higher level of need received specialist support such as one‐to‐one sessions with a physiotherapist, dietician or clinical psychologist, and participated in group exercise classes, whereas low‐need patients received home exercise programmes and online resources. All Active Together patients were offered support throughout their cancer treatment and up to 6 months of rehabilitation (3 months restorative and 3 months supportive) [24], although not all patients participated in rehabilitation. Detailed descriptions of the needs assessment and support offered are available elsewhere [23]. The length of prehabilitation varied depending on the treatment wait times. Between January 2022 (the inception of the service) and March 2024 (the end of the initial funding period), 425 patients received surgery for colorectal, lung or upper GI cancer after attending prehabilitation at Active Together. Patients who only received other forms of treatment (e.g. radical radiotherapy) but not surgery were not included in this analysis. 104 patients were excluded from further analysis, as they had not received at least two weeks of prehabilitation. Therefore, the results are an estimation of the average treatment effect on the treated.

#### 2.3.2 Comparator Groups

As Active Together is an NHS clinical service and not a research trial, a concurrent control group was not available. Patients who were referred to Active Together but declined to participate in the service (*N* = 96) were therefore used as a comparator group. A historical dataset comprising 3427 surgeries performed at Sheffield Teaching Hospitals NHS Trust between January 2017, and December 2021 was obtained to use as an additional comparator group. The use of historical data as a comparator group is an established approach used in single‐arm research trials [25].

### 2.4 Clinical Outcomes

The length of stay in hospital and the length of stay in critical care after surgery were obtained from hospital records. Additionally, the total number of days spent in hospital as an emergency readmission in the 90 days following surgery was included as a secondary outcome. Date of death was used to calculate one‐year survival rate.

### 2.5 Outliers and Missing Values

The mean and standard deviation of the historical data were used to exclude length of stay outliers more than 3.29 standard deviations above the mean, as done previously [26]. This equated to a length of stay of over 44 days. As the sample size in this study is relatively small, these outliers may have had a disproportionate effect on the results. Five Active Together outliers and 47 historical data outliers were removed. There were no length of stay outliers in the declined group.

There were no missing values in the final cohorts.

### 2.6 Matched Controls

To minimise bias related to variation in surgical complexity, Active Together patients were randomly matched to up to three historical controls [27], by procedure type and whether their tumour was malignant (*N* = 282) or benign (*N* = 23). Data were not available to match by disease progression or cancer stage; however, all patients were undergoing treatment with curative intent. A list of included surgical procedures is available in Appendix 2. A total of 11 Active Together patients with no appropriate match were excluded from further analysis. The final sample sizes for each group are shown in Table 1.

**Table 1:** Sample sizes and characteristics for each tumour and prehabilitation/non‐prehabilitation group.

Tumour group	Group	N	Mean age in years (SD)	Male sex (%)
Colorectal	Active Together	162	68.6 (10.0)	53.7
	Declined	31	68.0 (10.5)	51.6
	Historical	463	66.8 (11.1)	55.9
Lung	Active Together	81	67.8 (9.0)	46.9
	Declined	51	70.6 (8.2)*	47.1
	Historical	243	70.1 (9.0)*	51.9
Upper GI	Active Together	62	65.1 (9.6)	69.4
	Declined	14	68.6 (7.9)	78.6
	Historical	163	65.2 (10.0)	78.5
Total	Active Together	305	67.7 (9.7)	55.1
	Declined	96	69.5 (8.9)	53.1
	Historical	869	67.4 (10.5)	59.0

* Significantly different (*p* < 0.05) from Active Together group.

### 2.7 Cost Estimation

The estimated cost of the Active Together service was £712.86 per referred patient. This included support provided before and after surgery. This was calculated from the annual cost of running the service (including salary, premises, equipment, training, travel and miscellaneous costs) divided by the number of referrals received per year. The number of referrals received, rather than the number of patients who take up the offer, was used because there were costs associated with attempting to book patients in who eventually decline to join the service.

The hospital stay cost for surgical cancer treatment for each patient was calculated using the methods described by Arabadzhyan and colleagues [28]. The HRG4+ National Costs Grouper was used to assign HRG codes to each hospital spell and associated costs drawn from the 2022‐23 National Cost Collection data series.

### 2.8 Data Analysis

*T*‐test and Fishers' exact tests were used to compare the age and sex, respectively, between the Active Together group and the Declined and Historical groups. A Spearman's rank correlation was performed to test whether it was appropriate to include age as a covariate for length of stay analysis; however, there was no correlation between age and length of stay (rho = 0.003, *p* = 0.927).

The data were not normally distributed; therefore, the Mann–Whitney *U* test with a Benjamini–Hochberg correction for the two comparisons (to Declined and Historical groups) was used to compare healthcare resource use. *p* < 0.05 was considered statistically significant, and a Cohen's d effect size over 0.2 was considered meaningful [29]. Both means and medians are presented. The data were not normally distributed; however, the means are more clinically meaningful in this instance, as they reflect the total per‐patient cost incurred by the hospital.

A cost analysis was performed to determine whether Active Together was associated with increased overall treatment costs. Data were only available to compare with the Declined group. The analysis included the cost of the Active Together programme as well as the costs incurred during the initial hospital stay, including surgical procedures. Independent *t*‐tests were used to compare the treatment costs between the Active Together and Declined groups for each tumour group. A probability of cost‐saving is provided in line with conventional health economic methods [30], using 10,000 bootstrap samples. Where results show positive improvements in health outcomes, this can be considered to be a lower limit on the probability of cost‐effectiveness (i.e., a conservative estimate).

One‐year survival post‐surgery was analysed using a Cox proportional hazards regression model. *p* < 0.05 was considered statistically significant.

## 3 Results

### 3.1 Characteristics

Sample characteristics were not significantly different between groups, apart from in the lung cancer patients, where Active Together patients were significantly younger than the declined and historical groups (Table 1).

### 3.2 Prehabilitation Length

The average prehabilitation length (from the first appointment with Active Together to their first treatment) and the length of support before surgery is shown in Table 2.

**Table 2:** Mean (SD) Active Together prehabilitation length for each tumour group.

Tumour Group	Mean prehabilitation length in days (SD)	Mean days from first appointment to surgery (SD)
Colorectal (*N* = 162)	47.7 (34.7)	56.7 (52.7)
Lung (*N* = 81)	26.3 (14.2)	41.4 (30.8)
Upper GI (*N* = 62)	33.8 (14.4)	102.7 (43.0)
Total (*N* = 305)	36.8 (22.8)	62.2 (50.6)

### 3.3 Reasons for Declining

**Figure 2:**
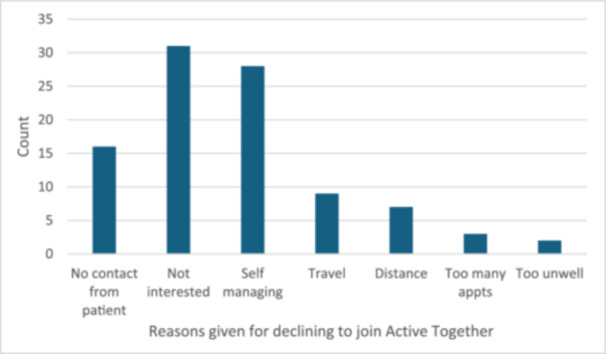
Reasons given by the ‘Declined’ group for not participating in the Active Together service. Appts; appointments.

The reasons for declining Active Together given by the declined group are shown in Figure 2. The most common reasons for declining were ‘Not interested’ (*N* = 31) and ‘Self‐managing’ (*N* = 28).

### 3.4 Length of Stay

There were no significant differences in length of stay between the groups, overall or for the tumour sub‐groups (Table 3). Historical colorectal patients had a significantly longer (+0.32 days) stay in critical care than Active Together patients. Similarly, the Declined colorectal group had a longer stay in critical care than Active Together colorectal patients, although this was not statistically significant (Table 3). Historical lung cancer patients spent significantly less time (−0.75 days) in critical care than Active Together patients.

**Table 3:** Hospital resource use after surgery for Active Together (AT), declined and historical patients.

Variable	Tumour group	Median (IQR)			Mean (SD)			Mean difference to AT† (95% CI)		*p* value (Effect size)	
		AT	Declined	Historical	AT	Declined	Historical	Declined	Historical	Declined	Historical
Length of stay in hospital	Colorectal	7 (2–22)	6 (0–33.5)	6 (2–30.5)	7.73 (5.22)	9.48 (9.14)	8.16 (7.18)	1.76 (−1.68– 5.20)	0.43 (−0.61–1.47)	0.810 (0.04)	0.810 (0.07)
	Lung	5 (2–15)	5 (2–18)	5 (2–16)	6.21 (3.68)	7.27 (6.15)	5.82 (3.92)	1.06 (−0.84–2.97)	−0.39 (−1.34–0.56)	0.947 (0.02)	0.408 (0.16)
	Upper GI	8 (5–23.5)	9 (6–17.5)	9 (5–33)	9.48 (5.20)	9.71 (3.63)	11.10 (7.09)	0.23 (−2.18–2.64)	1.61 (−0.09–3.32)	0.282 (0.28)	0.106 (0.31)
	Total	7 (2–22)	6 (2–29)	6 (2–29)	7.68 (4.97)	8.34 (7.03)	8.06 (6.65)	0.66 (−0.87–2.19)	0.38 (−0.34–1.09)	0.778 (0.03)	0.733 (0.06)
Length of stay in critical care	Colorectal	0 (0–0)	0 (0–0)	**0 (0–0)**	0.86 (1.70)	1.26 (2.24)	**1.18 (1.91)**	0.39 (−0.46–1.25)	**0.32 (0.00–0.64)**	0.507 (0.10)	**0.011** * **(0.26)**
	Lung	2 (2–3)	2 (2–2)	**2 (2–2)**	2.49 (1.77)	3.02 (4.37)	**1.74 (2.41)**	0.53 (−0.76–1.81)	**−0.75 (−1.25–−0.26)**	0.300 (0.23)	**< 0.0005** * **(0.61)**
	Upper GI	5 (4–6)	5.5 (4.5–7)	5 (5–6)	5.53 (3.46)	6.07 (3.08)	5.93 (3.25)	0.54 (−1.40–2.48)	0.40 (−0.61–1.41)	0.356 (0.24)	0.356 (0.20)
	Total	2 (0–2)	2 (2–2)	2 (2–2)	2.25 (2.83)	2.90 (3.91)	2.23 (2.96)	0.65 (−0.20–1.50)	−0.02 (−0.39–0.36)	0.376 (0.15)	0.664 (0.03)
Total days in hospital after readmission as an emergency, in the 90 days post‐surgery	Colorectal	0 (0–10.5)	0 (0–7)	0 (0–13. 5)	0.87 (3.17)	0.58 (2.16)	1.23 (4.73)	−0.30 (−1.30–0.71)	0.36 (−0.32–1.03)	0.607 (0.09)	0.545 (0.11)
	Lung	0 (0–15.5)	0 (0–7)	0 (0–11)	1.89 (6.36)	0.76 (1.97)	0.86 (3.08)	−1.14 (−2.71–0.44)	−1.04 (−2.56–0.49)	0.747 (0.08)	0.587 (0.14)
	Upper GI	0 (0–7)	0 (0–20.5)	0 (0–13)	0.79 (3.43)	2.36 (7.50)	1.73 (7.50)	1.56 (−2.84–5.97)	0.94 (−0.55–2.42)	0.571 (0.16)	0.42 (0.22)
	Total	0 (0–12.5)	0 (0–8)	0 (0–13)	1.14 (4.34)	0.96 (3.48)	1.22 (5.01)	−0.18 (−1.08–0.71)	0.08 (−0.54–0.70)	0.880 (0.02)	0.841 (0.06)

*Note:* Bold values indicate statistically significant.

Abbreviation: AT, Active Together.

* Statistically significant.

^†^ A positive value indicates a longer stay/more days in hospital compared to Active Together patients.

### 3.5 Hospital Readmissions

There were no significant differences in the total number of days spent in hospital after an emergency readmission in the 90 days post‐surgery (Table 3).

### 3.6 Surgery Stay Cost

There were no significant differences between the total cost of hospital stay and prehabilitation for Active Together patients compared to the declined group (Table 4). The probability of cost‐saving was in all cases greater than 50%.

**Table 4:** Total cost of treatment* for Active Together and Declined patients.

Tumour group	Mean (SD)		*p* value	Probability of cost‐saving
	Active Together	Declined		
Colorectal	£14,321.55 (£4267.95)	£14,581.99 (£6420.93)	0.829	58%
Lung	£16,789.06 (£4131.40)	£17,282.86 (£10,209.1)	0.743	60%
Upper GI	£23,081.70 (£8536.23)	£23,558.34 (£7068.09)	0.829	59%

* Including surgery and the cost of Active Together [31].

### 3.7 One‐Year Survival

Active Together patients were more likely to survive one year compared to the Declined group (Table 5). There were no significant differences in one‐year survival between the Active Together groups and the Historical groups.

**Table 5:** Survival rates and hazard ratios from Cox regression model.

Tumour group	Statistic	Active Together	Declined	Historical
Colorectal	1‐Year survival (95% CI)	97% (94%–100%)	86% (72%–100%)	95% (93%–97%)
	Hazard ratio (95% CI)	1	3.49 (0.78–15.6)	2.15 (0.76–6.07)
	*p* value		0.100	0.150
Lung	1‐Year survival (95% CI)	93% (87%–99%)	89% (80%–99%)	90% (86%–94%)
	Hazard ratio (95% CI)	1	1.32 (0.48–3.63)	1.13 (0.53–2.42)
	*p* value		0.600	0.750
Upper GI	1‐Year survival (95% CI)	91% (83%–100%)	**68% (45%–100%)**	85% (80%–91%)
	Hazard ratio (95% CI)	1	**3.88 (1.39–10.8)**	1.31 (0.61–2.83)
	*p* value		**0.010** *	0.490
Total	1‐Year survival (95% CI)	95% (92%–98%)	**85% (77%–93%)**	92% (90%–94%)
	Hazard ratio (95% CI)	1	**2.29 (1.19–4.40)**	1.43 (0.89– 2.30)
	*p* value		**0.013** *	0.140

*Note:* Bold values indicate statistically significant.

* Statistically significant. Hazard ratios for declined and historic data are calculated compared to Active Together patients.

## 4 Discussion

This study suggests that prehabilitation has a beneficial effect on healthcare resource use and one‐year survival. The small sample size means that the ability to detect significant differences in highly variable measures is limited; however, some meaningful differences (as defined by an effect size > 0.2) were found. For example, there was a meaningful improvement in length of stay for the upper GI Active Together patients, with declined and historical patients staying 0.23 and 1.61 days longer, respectively. This represents a meaningful reduction in NHS resources; however, a larger sample size is required to determine statistical significance.

The length of stay results are consistent with previous studies, which found nonsignificant reductions in length of stay after prehabilitation [14, 15, 16], although the magnitude of the reduction is smaller here. A similar service based in Greater Manchester, Prehab4Cancer, found length of stay was significantly shorter after prehabilitation for colorectal (−1.5 days) and lung (−0.4 days) cancer patients, but not significantly different for oesophageal (−0.2 days) cancer patients [31], compared to a combined historical, declined and not referred comparator group. Their non‐prehabilitation groups had a longer mean length of stay compared to the Sheffield historical patient cohort, suggesting a difference in regional hospital practice. Therefore, there may have been greater opportunity for improvement. In the Active Together analysis, all upper GI cancer groups had a shorter mean length of stay than the prehabilitation group in the Prehab4Cancer report. This demonstrates that the potential reductions in length of stay through prehabilitation may vary across hospitals, depending on current practice.

This study identified a significant difference in critical care days between the Active Together colorectal group (0.9 days) and the Declined group (1.2 days). Prehab4Cancer found a comparable difference in critical care days for their colorectal cancer patients (1.4 vs. 1.8 days for the prehabilitation and non‐prehabilitation groups, respectively) [31]. In this evaluation, there was a similar non‐significant trend for upper GI patients. Larger sample sizes are required to confirm these trends.

There was one result that suggested prehabilitation may increase hospital resource use, with Historical lung cancer patients spending significantly less time (−0.75 days) in critical care compared to Active Together patients. This difference may be partially explained by the inclusion of higher‐risk patients in the Active Together cohort (i.e. individuals who may not have previously been offered surgery due to poor baseline fitness). In some cases, patients were referred to Active Together with the goal of becoming fit enough for surgery through prehabilitation. As a result, the Active Together group may have included patients at greater risk of postoperative complications, potentially requiring longer hospital stays. Before the inception of Active Together, these patients would not have received surgery and, therefore, would not be included in the historical dataset.

The number of patients who were not eligible for surgery at the start of prehabilitation was not systematically recorded, although this has been reported by clinical teams anecdotally. Therefore, their inclusion in the final Active Together group is uncertain. A previous study on prehabilitation for lung cancer patients, however, found that only 41% patients were fit for surgery before prehabilitation, whereas after prehabilitation, 76% were fit for surgery [10]. This suggests that almost 60% of patients who were ineligible for surgery, became eligible after prehabilitation. Systematic recording of surgical eligibility would be useful to determine the potential confounding effect on the results.

There were no significant differences in the total number of days spent in hospital after an emergency readmission in the 90 days post‐surgery. This finding aligns with a previous meta‐analysis, which also found no significant difference in 30‐day hospital readmissions between prehabilitation and control groups in patients undergoing abdominal cancer surgery [13]. As there are many possible reasons for emergency readmission, this measure may not be specific enough to capture any differences due to improved preoperative fitness.

For all three tumour groups, the mean cost was higher for the declined group than the Active Together group, with average cost savings of £260, £494, and £477 for colorectal, lung and upper GI cancer patients, respectively. While not statistically significant, if the cost‐savings in this evaluation are extrapolated to a national level, it could save the NHS £19 million per year, based on the number of lung and gastrointestinal cancer cases [32] and the percentage that receive surgery [33]. These results are similar to a previous study that showed an average €140 saving for colorectal cancer patients 30‐day post‐operative hospital costs [17].

Given the improvement in fitness and wellbeing outcomes exhibited across patients in the Active Together group [34], the probabilities of cost‐effectiveness can be interpreted as a lower limit (i.e., conservative estimate) and give confidence that the intervention is more likely than not to be cost‐effective, assuming the declined group is a representative sample. This assumption is a key limiting factor in the interpretation of the cost‐saving results. The reasons given for declining Active Together suggest there are some key differences, with the most common reasons being ‘not interested’ and ‘self‐managing.’ This may reflect that patients do not believe they require structured support, or could indicate perceived barriers to participation, such as time constraints or a belief that existing self‐care strategies are sufficient. Only two patients gave the reason ‘too unwell,’ who were both lung cancer patients.

The Active Together model stratifies patients based on their level of need. The actual cost of delivering support, therefore, varies between individuals. For this evaluation, the same cost of Active Together (£712.86) has been allocated to each patient, as estimating the true cost would be very complex. This cost includes the whole Active Together pathway (prehabilitation, maintenance and rehabilitation), not just prehabilitation costs. The short‐term cost savings would be greater if only the cost of prehabilitation was included. There may be other long‐term cost savings that are not captured by this analysis, such as reduced reliance on primary care services.

Provision based on needs may be the most cost‐effective method, as those with higher initial needs will likely benefit the most, as suggested previously [18]. However, providing support to these patients typically incurs higher costs, due to the requirement for closer supervision and more intensive, in‐person support. As only Active Together patients underwent a needs assessment, it was not possible to compare the effectiveness of the intervention on sub‐groups of high or low needs patients in this evaluation.

Active Together is a clinician‐led service, which employs physiotherapists to provide physical activity support to patients with complex needs, alongside specialist fitness instructors for lower needs patients. In contrast, the leisure‐sector led model, delivered without clinical supervision is more likely to exclude patients with more complex health conditions. This high‐risk cancer population potentially have the most to gain from prehabilitation, as they face greater risks of treatment‐related complications and functional decline. Additionally, a prehabilitation service for ovarian cancer patients based in Wales found that engagement improved when they moved to a physiotherapy‐based service, rather than an exercise‐referral service, suggesting that patients placed higher importance on a clinician‐led service [35]. The creation of a service that is delivered predominantly by allied health professionals allows for the inclusion of the most complex and high‐risk patients, and may improve overall engagement, but requires appropriate resource with cost implications. The optimal prehabilitation model is yet to be determined.

The type of surgery may also influence whether the intervention is cost‐effective. It has been suggested that prehabilitation may be more cost‐saving when the surgery type is particularly aggressive [36]. Larger sample sizes would allow for differences between surgery types and complexity to be examined.

For all tumour groups, the percentage of patients surviving one‐year was highest in the Active Together group, compared to the Declined or Historical groups. This provides promising evidence of the positive effect of prehabilitation and rehabilitation on overall survival; however, the historical comparisons may be confounded by improvements to cancer treatments. Similarly, declined groups may have been more unwell or from disadvantaged backgrounds, and may receive different treatment from healthcare professionals who were aware of whether patients are participating in the service. Longer term examinations of overall and disease‐free survival are necessary to build on this area, across cancer types.

### 4.1 Statistical Power

This evaluation was performed using all the available data from the evaluation time period. The limited sample sizes mean that the statistical tests are underpowered and, therefore, unlikely to demonstrate a statistically significant result. Given the standard deviation, in order to find a statistically significant difference if length of stay is different by one between the groups, 3056 patients would be required in total. A future evaluation of Active Together services across Yorkshire is planned, which will greatly increase the power. Nevertheless, this paper provides important and timely insights into the potential economic impact of services such as Active Together.

### 4.2 Limitations

This evaluation was limited by the available data. A relatively small cohort of patients had completed at least two weeks of prehabilitation prior to surgery. The prehabilitation support offered varied based on patient needs and preferences, and adherence was not measured; therefore, the intervention ‘dose’ cannot be quantified. Additionally, confounding factors such as comorbidities, cancer stage and baseline functional status were not available.

As an evaluation rather than a randomised, blinded research study, healthcare professionals were aware of patients' participation in prehabilitation, which could have affected the treatment they received. The comparator groups may be meaningfully different in ways that are not captured by this evaluation, and changes to hospital practices over time, including changes over Covid‐19 lockdowns, could affect the comparison to historical data. These factors will limit the generalisability of the results.

### 4.3 Further Study

The Active Together service has now expanded across Yorkshire, delivering across eight NHS trusts with further plans for expansion. A larger economic and survival evaluation will be conducted, which will allow for more robust conclusions to be drawn. Where possible, additional data will be collected to address the limitations above.

## 5 Conclusions

Patients that received prehabilitation in a real‐world setting did not have higher overall healthcare costs compared to those that declined the service and had a higher one‐year survival rate. Combined with the positive impact on patient outcomes, these results build upon previous research demonstrating the positive impact of integrating prehabilitation services like Active Together into standard pre‐surgical care pathways for cancer patients. Larger‐scale evaluations and clinical comparator studies are needed to confirm the positive trends regarding length of stay and one‐year survival and understand the impact on access to surgery.

## Author Contributions

**Kerry Rosenthal:** methodology, formal analysis, data curation, writing – original draft. **Nik Kudiersky:** methodology, writing – review and editing. **Gabriella Frith:** conceptualisation, methodology, writing – review and editing, funding acquisition. **Gail Phillips:** project administration. **Carol Keen:** methodology, investigation, resources. **Daniel Howdon:** methodology, formal analysis, writing – review and editing. **Shaun Barratt:** software, data curation. **Diana M. Greenfield:** conceptualisation, writing – review and editing. **Gary H. Mills:** conceptualisation, writing – review and editing. **Anna Myers:** conceptualisation, methodology, writing – review and editing, funding acquisition. **Liam Humphreys:** conceptualisation, methodology, writing – review and editing, funding acquisition. **Robert Copeland:** conceptualisation; methodology, supervision, funding acquisition, resources.

## Conflicts of Interest

The authors declare no conflicts of interest.

## Supporting information

Supporting File 1

Supporting File 2

## Data Availability

The data that support the findings of this study are available from Sheffield Teaching Hospitals NHS Trust. Restrictions apply to the availability of these data, which were used under license for this study.

## References

[1] C. Li , F. Carli , L. Lee , et al., “Impact of a Trimodal Prehabilitation Program on Functional Recovery After Colorectal Cancer Surgery: A Pilot Study,” Surgical Endoscopy 27 (2013): 1072–1082. 23052535 10.1007/s00464-012-2560-5

[2] J. Wang , C. Wei , S. L. Tucker , et al., “Predictors of Postoperative Complications After Trimodality Therapy for Esophageal Cancer,” International Journal of Radiation Oncology*Biology*Physics 86 (2013): 885–891. 10.1016/j.ijrobp.2013.04.006 PMC3786201 23845841

[3] P. Kirchhoff , P. A. Clavien , and D. Hahnloser , “Complications in Colorectal Surgery: Risk Factors and Preventive Strategies,” Patient Safety in Surgery 4 (2010): 5. 20338045 10.1186/1754-9493-4-5 PMC2852382

[4] D. Coderre , P. Brahmbhatt , T. L. Hunter , and J. Baima , “Cancer Prehabilitation in Practice: The Current Evidence,” Current Oncology Reports 24 (2022): 1569–1577. 35788874 10.1007/s11912-022-01304-1

[5] C. Fleurent‐Grégoire , N. Burgess , D. I. McIsaac , et al., “Towards a Common Definition of Surgical Prehabilitation: A Scoping Review of Randomised Trials,” British Journal of Anaesthesia 133 (2024): 305–315. 38677949 10.1016/j.bja.2024.02.035 PMC11282475

[6] N. L. Stout , J. Baima , A. K. Swisher , K. M. Winters‐Stone , and J. Welsh , “A Systematic Review of Exercise Systematic Reviews in the Cancer Literature,” PM & R 9 (2017): S347–S384. 28942909 10.1016/j.pmrj.2017.07.074 PMC5679711

[7] P. Cormie , M. Atkinson , L. Bucci , et al., “Clinical Oncology Society of Australia Position Statement on Exercise in Cancer Care,” Medical Journal of Australia 209 (2018): 184–187. 29719196 10.5694/mja18.00199

[8] D. R. Kaye , C. Schafer , S. Thelen‐Perry , et al., “The Feasibility and Impact of a Presurgical Exercise Intervention Program (Prehabilitation) for Patients Undergoing Cystectomy for Bladder Cancer.” Urology (Ridgewood, N.J.) (2020). 145, 106–112. 10.1016/j.urology.2020.05.104 32739310

[9] R. Perry , G. Herbert , C. Atkinson , et al., “Pre‐Admission Interventions (Prehabilitation) to Improve Outcome After Major Elective Surgery: A Systematic Review and Meta‐Analysis,” BMJ Open 11 (2021): e–e050806. 10.1136/bmjopen-2021-050806 PMC8487197 34593498

[10] I. Goldsmith , G. Chesterfield‐Thomas , and H. Toghill , “Pre‐Treatment Optimization With Pulmonary Rehabilitation in Lung Cancer: Making the Inoperable Patients Operable,” EClinicalMedicine 31 (2021): 100663. 33554075 10.1016/j.eclinm.2020.100663 PMC7846708

[11] S. C. Buttigieg , L. Abela , and A. Pace , “Variables Affecting Hospital Length of Stay: A Scoping Review,” Journal of Health Organization and Management 32 (2018): 463–493. 29771210 10.1108/JHOM-10-2017-0275

[12] C. Treanor , T. Kyaw , and M. Donnelly , “An International Review and Meta‐Analysis of Prehabilitation Compared to Usual Care for Cancer Patients,” Journal of Cancer Survivorship 12 (2018): 64–73. 28900822 10.1007/s11764-017-0645-9

[13] J. L. Waterland , O. McCourt , L. Edbrooke , et al., “Efficacy of Prehabilitation Including Exercise on Postoperative Outcomes Following Abdominal Cancer Surgery: A Systematic Review and Meta‐Analysis,” Frontiers in Surgery 8 (2021): 628848. 33816546 10.3389/fsurg.2021.628848 PMC8017317

[14] J. Dronkers , H. Lamberts , I. Reutelingsperger , et al., “Preoperative Therapeutic Programme for Elderly Patients Scheduled for Elective Abdominal Oncological Surgery: A Randomized Controlled Pilot Study,” Clinical Rehabilitation 24 (2010): 614–622. 20530651 10.1177/0269215509358941

[15] K. Valkenet , J. C. A. Trappenburg , J. P. Ruurda , et al., “Multicentre Randomized Clinical Trial of Inspiratory Muscle Training Versus Usual Care Before Surgery for Oesophageal Cancer,” British Journal of Surgery 105 (2018): 502–511. 29603130 10.1002/bjs.10803

[16] M. Kaibori , M. Ishizaki , K. Matsui , et al., “Perioperative Exercise for Chronic Liver Injury Patients With Hepatocellular Carcinoma Undergoing Hepatectomy,” American Journal of Surgery 206 (2013): 202–209. 23374372 10.1016/j.amjsurg.2012.07.035

[17] C. R. Sabajo , D. W. G. Ten Cate , M. H. M. Heijmans , C. T. G. Koot , L. V. L. Van Leeuwen , and G. D. Slooter , “Prehabilitation in Colorectal Cancer Surgery Improves Outcome and Reduces Hospital Costs,” European Journal of Surgical Oncology 50 (2023), 10.1016/j.ejso.2023.107302. 38043359

[18] Y. Ke , R. R. G. Ng , S. Elangovan , et al., “Prehabilitation Programs—A Systematic Review of the Economic Evidence,” Frontiers in Medicine 10 (2023), 10.3389/fmed.2023.1281843. PMC10722222 38105890

[19] T. Rombey , H. Eckhardt , J. Kiselev , J. Silzle , T. Mathes , and W. Quentin , “Cost‐Effectiveness of Prehabilitation Prior to Elective Surgery: A Systematic Review of Economic Evaluations,” BMC Medicine 21 (2023): 265. 37468923 10.1186/s12916-023-02977-6 PMC10354976

[20] M. Trépanier , E. M. Minnella , T. Paradis , et al., “Improved Disease‐Free Survival After Prehabilitation for Colorectal Cancer Surgery,” Annals of Surgery 270 (2019): 493–501. 31318793 10.1097/SLA.0000000000003465

[21] H. C. van der Hulst , J. M. van der Bol , E. Bastiaannet , J. E. A. Portielje , and J. W. T. Dekker , “The Effect of Prehabilitation on Long‐Term Survival and Hospital Admissions in Older Patients Undergoing Elective Colorectal Cancer Surgery,” European Journal of Surgical Oncology 50 (2024): 108244. 38452716 10.1016/j.ejso.2024.108244

[22] Y. Liu , X. Chen , and L. Zou , “Boosting Recovery before Surgery: The Impact of Prehabilitation on Upper Gastrointestinal Cancer Patients—A Quantitative Comparative Analysis,” PLoS One 20 (2025): e0315734. 40100884 10.1371/journal.pone.0315734 PMC11918424

[23] L. Humphreys , A. Myers , G. Frith , et al., “The Development of a Multi‐Modal Cancer Rehabilitation (Including Prehabilitation) Service in Sheffield, UK: Designing the Active Together Service,” Healthcare 12 (2024): 742. 38610164 10.3390/healthcare12070742 PMC11011813

[24] A. Myers , L. Humphreys , M. Thelwell , et al., “Embedding Multimodal Rehabilitation Within Routine Cancer Care in Sheffield—The Active Together Service Evaluation Protocol,” Journal of Physical Activity & Health 21 (2024): 1080–1091. 39151907 10.1123/jpah.2023-0622

[25] C. Mack , J. Christian , E. Brinkley , E. J. Warren , M. Hall , and N. Dreyer , “When Context Is Hard to Come by: External Comparators and How to Use Them,” Therapeutic Innovation & Regulatory Science 54 (2020): 932–938. 32557316 10.1007/s43441-019-00108-z

[26] F. I. Mowbray , S. M. Fox‐Wasylyshyn , and M. M. El‐Masri , “Univariate Outliers: A Conceptual Overview for the Nurse Researcher,” Canadian Journal of Nursing Research 51 (2019): 31–37. 10.1177/0844562118786647 29969044

[27] D. E. Banks , R. Shi , D. F. Timm , et al., “Decreased Hospital Length of Stay Associated With Presentation of Cases at Morning Report With Librarian Support,” Journal of the Medical Library Association: JMLA 95 (2007): 381–387. 17971885 10.3163/1536-5050.95.4.381 PMC2000787

[28] A. Arabadzhyan , A. Castelli , J.M. Gaughan , and M.J. Chalkley, “Productivity of the English National Health Service: 2021/22 Update,” Discussion Paper, CHE Research Paper Series, Centre for Health Economics, University of York (2024), 10.15124/yao-4n1s-cc89.

[29] E. M. Winter , G. A. Abt , and A. M. Nevill , “Metrics of Meaningfulness as Opposed to Sleights of Significance,” Journal of Sports Sciences 32 (2014): 901–902. 24650348 10.1080/02640414.2014.895118

[30] K. Claxton , “The Irrelevance of Inference: A Decision‐Making Approach to the Stochastic Evaluation of Health Care Technologies,” Journal of Health Economics 18 (1999): 341–364. 10537899 10.1016/s0167-6296(98)00039-3

[31] N. H. S. South , Central and West Commissioning Support Unit. (Prehab4Cancer Evaluation, 2022).

[32] Cancer Research UK , Statistics by Cancer Group, 2024, accessed January 22, 2025, https://www.cancerresearchuk.org/.

[33] National Disease Registration Service , Treatment by Demographic Factors, 2024, accessed January 22, 2025, http://nhsd‐ndrs.shinyapps.io/.

[34] N. Kudiersky , K. Rosenthal , G. Frith , et al., “A Service Evaluation of the Active Together Multi‐Modal Cancer Prehabilitation and Rehabilitation Service,” medRxiv (2025), In press.

[35] J. C. McMullan , C. Smith , R. Jones , et al., “All Wales Ovarian Cancer Prehabilitation Project (AWOCPP),” BMJ Open Quality 14 (2025): e002770. 10.1136/bmjoq-2024-002770 PMC12083423 40000106

[36] R. Risco , R. González‐Colom , M. Montané‐Muntané , et al., “Actionable Factors Fostering Health Value Generation and Scalability of Prehabilitation,” Annals of Surgery 278 (2022): e217–e225. 35968894 10.1097/SLA.0000000000005662 PMC10321511

